# Editorial: Global excellence in rheumatology: Africa

**DOI:** 10.3389/fmed.2023.1201020

**Published:** 2023-04-28

**Authors:** Girish M. Mody, Mohammed Tikly, Najia Hajjaj-Hassouni

**Affiliations:** ^1^Department of Rheumatology, University of KwaZulu-Natal, Durban, South Africa; ^2^Division of Rheumatology, University of the Witwatersrand, Johannesburg, South Africa; ^3^Faculty of Medicine, International University of Rabat, Salé, Morocco

**Keywords:** Africa, tuberculosis, systemic lupus erythematosus, idiopathic inflammatory myopathies, women, rheumatologists, systemic juvenile idiopathic arthritis, tocilizumab

The United Nations estimates that in 2023 over 1.4 billion people, or 16.7% of the world's population, reside in Africa. Africa faces many healthcare challenges due to multiple factors, including socioeconomic factors, poor infrastructure, and lack of human resources. Much of its health resources are devoted to combating often lethal communicable diseases such as tuberculosis, HIV infection, and malaria. It is not surprising, therefore, that the subspecialty of rheumatology has been a late bloomer within the wider internal medicine fraternity (1). A recent survey of the 54 African countries, with responses from 44 countries (81%), showed that most of the 2,970 rheumatologists on the continent are based in North African countries, while 12 (27%) countries have between one and 10 rheumatologists, and 17 (39%) have no rheumatologists (2). The current themed series, Global excellence in Rheumatology: Africa, is a timely reminder of the challenges and steady progress of rheumatology in Africa.

Hmamouchi and Adebajo recognize the contributions of outstanding African women in rheumatology amongst the many women who have provided excellent rheumatology care to patients in many countries for decades. The vision and foresight of Professors Najia Hajjaj-Hassouni and Aicha Ladjouze over more than four decades have played a key role in the establishment of national rheumatology associations and associated scientific congresses in Morocco and Algeria, respectively. Both have served on the African League of Associations for Rheumatology (AFLAR) executive as President-Elect and then President of AFLAR.

Dr. Dey and Professor Migowa, on the other hand, represent the dynamism of the present and future of rheumatology on the continent. Dzifa Dey, the current AFLAR President, having just completed her term as Secretary-General, has been instrumental in recent years for raising the profile and growth of AFLAR. Despite her clinical and academic commitments in Ghana, her excellent organizational skills have led to greater awareness and recognition of African rheumatology in Africa and globally. Angela Migowa in Kenya and Professor Chris Scott in South Africa are very much the nexus of African pediatric rheumatology. Angela is the founder and current President of PAFLAR (pediatric arm of AFLAR). Her energy, vision, and motivation have led to increased awareness of childhood rheumatic diseases through regular scientific meetings and congresses that attract audiences from the continent and beyond. Finally, it would be remiss of us not to recognize the work and contributions of the late Dr. Marie Louise Doulla of Cameroon, who tragically passed away in 2018 during her term as President-Elect of AFLAR.

Nada et al. report their positive experience of a 1-year open-labeled study with tocilizumab in the treatment of systemic juvenile idiopathic arthritis (sJIA) in patients who were treated with conventional therapy. Of the 65 Egyptian children with sJIA included in the study, just over a third achieved clinically inactive disease, and a further quarter achieved minimal disease activity. Less than 10% had a hypersensitivity reaction to the drug. Much like in previous studies, children ≤7 years, a disease duration ≤3 years, lower disease activity, and higher serum ferritin, and the systemic manifestations had a more favorable outcome. These findings provide further reassuring evidence of the effectiveness and safety of tocilizumab for a potentially disabling and fatal condition.

Tuberculosis (TB) remains a major concern in Africa, despite the WHO's End TB Strategy that was adopted in 2014. South Africa belongs to the 30 high TB burden countries with more than 500 cases/100,000 people. The study by Al-arbi et al. of TB in systemic lupus erythematosus (SLE) patients is a stark reminder of the impact of TB in systemic autoimmune rheumatic diseases (SARDs). The treatment and prognosis of SLE has improved substantially in recent decades, but this records review study of a multi-ethnic population at a single academic center in Durban, South Africa, found that of the 72 (14.1%) out of the 512 SLE patients that had contracted TB, the majority (58%) had extrapulmonary TB (E-PTB). The ethnic differentiation of TB prevalence, highest in patients of mixed ancestry and Black Africans, while there is no prevalence in the White population, reflects in part the persistent inter-ethnic socioeconomic differences in South Africa. Compared to the control SLE patients without TB, TB patients had higher disease activity and cumulative prednisone use over the preceding 3 months. Compared to the patients with pulmonary TB only, the EPTB patients had higher disease activity, more renal involvement, and were more likely to have IV methylprednisolone (IVMP) or mycophenolate mofetil therapy. Notwithstanding the limitations of the study as indicated by the authors, the study provides further evidence of the burden of TB on SLE patients in Africa.

Idiopathic inflammatory myopathies (IIM) are a group of rare SARDs for which there is a paucity of data in Africa. Birch et al. undertook a retrospective records review study of the clinical spectrum and outcomes of 94 patients fulfilling the Bohan and Peter criteria for IIM and attending a tertiary rheumatology center in South Africa. Most were indigent Black people mainly with dermatomyositis (DM) and polymyositis (PM). Despite an earlier onset of disease compared to patients in developed countries, the clinical, biological, and therapeutical data were similar. The predominance of DM could be explained by high UV light exposure in tropical and subtropical regions. Dysphagia, which requires pulse IVMP therapy, was considered a strong indicator of severity, more often reported in PM than DM patients. The Jo-1 antibody test was present in one-fifth of patients and occurred more often in patients with PM and those with ILD; this latter finding is congruent with the reports from the EuroMyositis registry (25–30%) (3). This antibody occurred most frequently in association with the anti-synthetase syndrome. Malignancies were documented in only three patients (3.2%). Patients were treated according to the current international standard of care, and the drug therapy comprised either oral corticosteroids or IVMP, with methotrexate and azathioprine as corticosteroid-sparing agents. Despite its significant limitations, this study delineates the features of these rare conditions in Black Africans.

These papers provide a glimpse of the steady growth of African rheumatology. With advances and improved access to laboratory technologies, African rheumatology will make a greater contribution to the global quest to conquer rheumatic musculoskeletal diseases.

## Author contributions

GM, MT, and NH-H: conception, review of information and preparation of part of editorial, and review of final paper. All authors contributed to the article and approved the submitted version.
